# Fast-Growing Bacterial Cellulose with Outstanding Mechanical Properties via Cross-Linking by Multivalent Ions

**DOI:** 10.3390/ma13122838

**Published:** 2020-06-24

**Authors:** Andrea Knöller, Marc Widenmeyer, Joachim Bill, Zaklina Burghard

**Affiliations:** 1 Institute for Materials Science, University of Stuttgart, 70569 Stuttgart, Germany; Andrea.Knoeller@Hahn-Schickard.de (A.K.); marc.widenmeyer@mr.tu-darmstadt.de (M.W.); bill@imw.uni-stuttgart.de (J.B.); 2 Institute for Micro Assembly Technology of the Hahn-Schickard-Gesellschaft e.V., 70569 Stuttgart, Germany; 3 Department of Materials and Earth Science, Materials and Resources, Technical University of Darmstadt, 64287 Darmstadt, Germany

**Keywords:** kombucha, bacterial cellulose, membranes, cross-linking, mechanical properties

## Abstract

Bacterial cellulose is an organic product of certain bacterias’ metabolism. It differs from plant cellulose by exhibiting a high strength and purity, making it especially interesting for flexible electronics, membranes for water purification, tissue engineering for humans or even as artificial skin and ligaments for robotic devices. However, bacterial cellulose’s naturally slow growth rate has limited its large-scale applicability to date. Titanium (IV) bis-(ammonium lactato) dihydroxide is shown to be a powerful tool to boost the growth rate of bacterial cellulose production by more than one order of magnitude and that it simultaneously serves as a precursor for the Ti^4+^-coordinated cross-linking of the fibers during membrane formation. The latter results in an almost two-fold increase in Young’s modulus (~18.59 GPa), a more than three-fold increase in tensile strength (~436.70 MPa) and even a four-fold increase in toughness (~6.81 MJ m^−^³), as compared to the pure bacterial cellulose membranes.

## 1. Introduction

Cellulose is the most abundant organic polymer on earth. Typically extracted from plants, it cannot only find use as paper, but also as a mechanically stable matrix of numerous hybrid materials [1,2]. Xu et al. fabricated a cellulose/polysulfonamide composite membrane as separator for lithium-ion batteries, which exhibited enhanced wettability and heat tolerance due to the presence of the hydrophilic and inherently stable cellulose [3]. In addition, cellulose’s transparent nature and its mechanical flexibility give rise to self-supporting optoelectrical switching devices [4] and nonlinear optical applications [5]. Finally, owing to its low toxicity, cellulose is likewise considered in many biomedical applications such as drug delivery [6] and tissue engineering [7,8,9].

**Figure 1:**
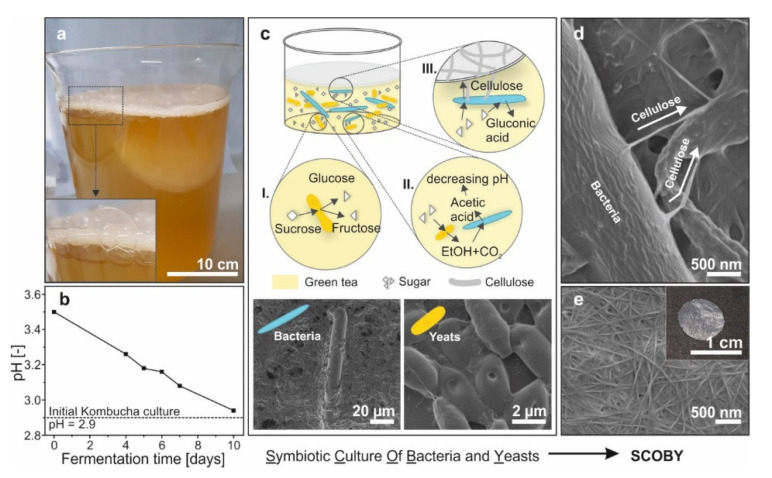
(**a**) Kombucha tea with a floating cellulose pellicle with bubbles of gaseous CO_2_ below, as magnified in the inset. (**b**) During fermentation, the pH steadily decreases, which originates from the metabolic activities of the yeasts and bacteria, as schematically depicted in (**c**): I. Initially, the yeasts split the sucrose into fructose and glucose. II. The yeasts further process both monosaccharides, which results in a release of EtOH and CO_2_. These products are then converted to acetic acid by the bacteria. III. The bacteria also directly process both monosaccharides to produce cellulose fibers. Such fibers form an entangled network, which floats on the surface of the tea. In addition, the glucose is processed into gluconic acid. Corresponding SEM images of (**d**) the cellulose production of the bacteria and (**e**) the entangled fiber network of the resulting bacterial cellulose membrane in the inset.

While plant cellulose apparently holds great technological potential in various fields of application, its acquisition typically involves deforestation coupled with chemical-intensive and energy- and time-consuming treatments of the cut wood, including biological and chemical pre-treatments, mechanical disintegration, as well as post-treatments [10]. Alternative to this conventional plant cellulose production, certain species of bacteria [11] can produce pristine cellulose fibers in a more eco-friendly way. These bacteria are ingredients of an ancient Asian beverage named Kombucha (Figure 1).

Kombucha is typically made from sweetened black or green tea, which is inoculated with a symbiotic culture of bacteria and yeasts (SCOBY) (Figure 1a). After adding the SCOBY to the tea, the fermentation process begins, which leads to a steady decrease in the tea’s pH (Figure 1b). This fermentation is the result of several metabolic activities of the microorganisms in the tea [12], as schematically depicted in Figure 1c. In a first step, the sucrose is split into fructose and glucose by the yeasts. Both monosaccharides can be further processed by bacteria as well as by the yeasts. In the case of the latter, ethanol (EtOH) and CO_2_ is released, whereas bubbles of gaseous CO_2_ can get trapped below the floating bacterial cellulose pellicle, as displayed in Figure 1a. Both products are then used by the bacteria to produce acetic acid, which results in the observed decrease in pH. Alongside this, the bacteria can take up glucose to either produce gluconic acid, which further decreases the pH, or the desired bacterial cellulose (BC). The BC production itself occurs extracellular. The bacteria secrets bundles of polymerized cellulose microfibrils from their cell walls (Figure 1d) [13]. These cellulose bundles finally form an entangled fiber network (BC pellicle), in which the majority of the bacteria are immobilized [11]. In general, BC has a fiber network structure and is composed of glucose monomers connected by β(1–4) glycosidic linkages in chain form, with a high degree of polymerization [14]. Subsequently, exposing the BC pellicle to NaOH removes the microorganisms [15], leaving purified cellulose behind (Figure 1e). The concentration of bacteria in the medium as well as the amount of produced BC depends on the species and strains engaged in the reactions and the metabolic activity of the culture and the environmental conditions, including the type of culture medium and sugar [16] as well as temperature and pH [17].

This study addresses controlling the growth of the BC fibers alongside their simultaneous cross-linking via multivalent ions. While the first aims at speeding up the naturally slow pellicle growth, the latter is expected to significantly enhance the resulting membranes’ mechanical properties, analogous to what was found for graphene oxide membranes [18]. In this context, Titanium (IV) bis-(ammonium lactato) dihydroxide (Ti-BALDH) has great potential. This low-cost and water-based precursor for soft chemistry and bioinspired reactions allows the tea’s culture medium to be modified along with its pH (see Supplemental Materials Figure S1) and can serve as precursor for in situ cross-linking [19]. Therefore, combining ancient Kombucha tea (fermented stock solution) with modern Ti-BALDH is expected to enable an eco-friendly and time efficient production of BC membranes with mechanical stability, which opens the way for application in, e.g., robotics, medicine and water purification.

## 2. Materials and Methods

### 2.1. Preparing the Kombucha Stock Solution and the Kombucha Membranes

The purchased Kombucha culture (Bio-Kombuchapilz, Fairment, Berlin, Germany) contained 300 ml fermented liquid with a mixture of acetic acid bacteria (including *Komagataeibacter xylinus)* and yeasts (including *Saccharomyces ludwigii*) as well as a ~1 cm thick SCOBY pellicle. Titanium (IV) bis-(ammonium lactato) dihydroxide (Ti-BALDH), 50 wt.% in H_2_O (pH = 7.4), which served as precursor for the cross-linking, was purchased from Sigma-Aldrich (Darmstadt, Germany). For preparing the Kombucha stock solution, a sweetened green tea was prepared by steeping 13 tea bags (Green tea, Meßmer, 1.75 g each) for 10 min in 1 L boiling deionized water (DI). After removing the tea bags, 216 g sucrose were dissolved in the hot tea. Subsequently, 1.7 L cold deionized water was added to the sweet tea, which then cooled to room temperature. Finally, the purchased 300 mL fermented Kombucha culture and the pellicle were poured into the cold tea. The stock solution was obtained after fermenting this mixture for 10 days at room temperature. To prepare the Kombucha membranes, 120 mL of stock solution were diluted with 40 mL deionized water (volume ratio of 3:1). The obtained 160 mL solution was then refermented for another 7 days (Supplementary Materials Figures S1 and S2). The newly formed pellicle was harvested and washed by immersing it in deionized water for 48 h. Subsequently, the pellicles (Ti0) were dried free-standing (Supplementary Materials Figure S3). To prepare the Ti-cross-linked Kombucha membranes, 120 mL of stock solution were diluted with 40 mL Ti-BALDH precursor solution of different concentrations (0.1, 0.2 and 1 M) to obtain 160 mL of mixed solution with the resulting concentrations of 50, 100 and 250 mM, respectively. It has to be noted that by adding the Ti-BALDH solution, which itself has an initial pH of 7.4, the pH of the mixed solutions significantly increases (Supplementary Materials Figure S1). Analogous to the pure Kombucha membranes, the mixture was refermented for 7 days at room temperature (Supplementary Materials Figures S1 and S2), and the pellicle was harvested and washed by immersing it in deionized water for 48 h. Likewise, the pellicles (Ti50, Ti100 and Ti250) were dried free-standing at room temperature, which took up to one week, depending on the thickness of the pellicles (Supplementary Materials Figure S3). In order to remove the microorganisms from the membranes, they were post-treated by immersing them in 0.25 M NaOH at room temperature for 48 h, then washed in DI water and finally dried free-standing [15]. Subsequent annealing in an oven at 110 °C in ambient atmosphere for 1 h ensured the removal of residual water.

### 2.2. Characterization Methods

The thickness of the dried membranes was measured inductively using a self-built device, which converts a height difference of 1 µm into a measurable voltage of 1 V with the help of an HBM measuring amplifier (Supplementary Figure S4). The measured thicknesses were verified and visualized using SEM Carl Zeiss (Zeiss, Jena, Germany) cross-section analysis. The sample preparation for SEM investigation is presented in Supplementary Materials Figure S5. The SEM Zeiss Merlin (Zeiss, Oberkochen, Germany) was operated at 1.5 kV and 53 pA with a working distance of about 3 mm. Imaging was conducted with an in-lens detector for both cross-section and surface. FTIR absorption spectra were recorded with a Bruker Tensor 27 FT-IR (Bruker, Karlsruhe, Germany) equipped with an A225/Q Platinum Diamond Micro-ATR cell and an OPUS data collection program. The Ti content was determined using a Spectro Ciros CCD ICP-OES instrument (SPECTRO Analytical Instruments, Kleve, Germany). For the mechanical characterization of the samples nanotensile tests were conducted on narrow membrane strips (~0.2 mm), which were glued onto cardboard sample holders, yielding a gauge length of 10 mm. The used UTM 150 (Keysight, Böblingen, Germany), which was equipped with a CDA control unit and NanoSuite software, has a load limit of 0.5 N and a load resolution of 50 nN. Experiments were carried out 15 times on all samples with the same strain rate of 1 × 10^−3^ mm s^−1^ until fracture. As the actual pure BC samples (Ti0) were too thin and fragile for proper handling, their nanotensile tests were conducted on membranes with a thickness of 6.1 ± 0.4 µm, which could be achieved by increasing the growth time.

## 3. Results and Discussion 

**Figure 2:**
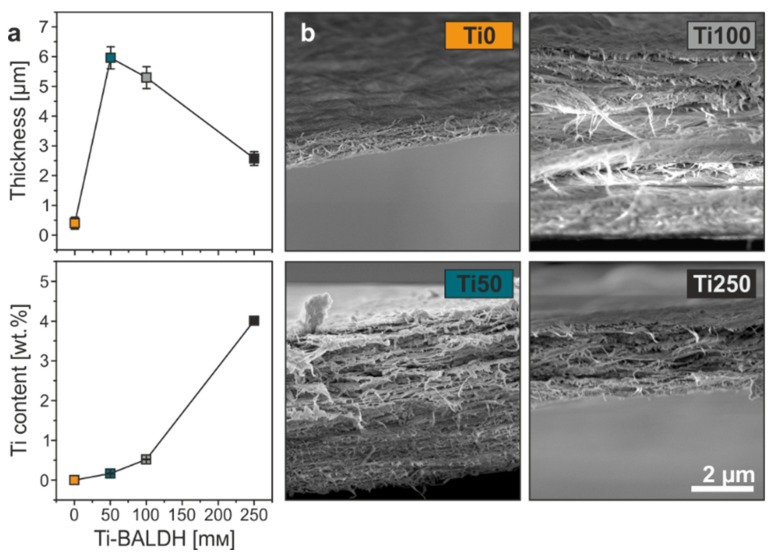
(**a**) Thickness and Ti content as a function of Ti-BALDH concentration in the solutions. (**b**) Corresponding SEM micrographs of the final membranes’ cross-sections. The displayed scale bar is valid for all four micrographs.

One pure Kombucha tea (0 mM Ti-BALDH) as well as three different Ti-BALDH-containing teas were prepared (50, 100 and 250 mM Ti-BALDH) from the fermented stock solution (see Supplemental Materials Figure S2a), and their resulting membranes were labeled as Ti0, Ti50, Ti100 and Ti250, respectively. Inductive thickness measurements (Figure 2a) and SEM cross-sectional investigations (Figure 2b) revealed that the membranes strongly differ in thickness (see also Supplemental Materials Table S1). The pure BC (Ti0) membranes exhibited by far the lowest value (0.4 ± 0.2 µm), followed by the membranes Ti250 (2.6 ± 0.2 µm), which grew in the tea with the highest Ti-BALDH concentration. In contrast, Ti50 and Ti100 were by far the thickest membranes with 6.0 ± 0.4 µm and 5.3 ± 0.4 µm, respectively. Therefore, these two membranes were produced with a rate that was more than one order of magnitude faster than the one of the pure BC. The non-linear trend in thickness is assumed to originate from two counteracting influences: (1) an increased pH can enhance the cell growth and cellulose production, while (2) the SCOBY is exposed to an unknown additive, which could unbalance the biological system resulting in cellulose production inhibition. Indeed, in the case of Ti0, which grew in the pure, fermented tea at a pH of about only 3 (Supplemental Materials Figure S1), the bacteria appear to be poorly active, producing only a little cellulose, thus resulting in a low yield. A direct comparison of membranes before and after post-treatment (Supplementary Materials Figure S2 and Table S1) support this statement. Removing the microorganisms reduced the thickness by 53.5%, meaning about half of the as-prepared membrane was composed of the microorganisms. Ti50 and Ti100 grew at a pH of about 4 and 4.2, respectively (Supplementary Materials Figure S1). This increased pH seems to be the key to (re)activate the bacteria, thus boosting the BC production. Complementarily, the comparison between as-prepared and post-treated membranes reveals a reduction in thickness by only 9.7 and 15.6%, resulting in much higher BC yields. Hwang et al. [17] found that a pH of 5.5 is preferred for the cell growth and cellulose production. However, this finding is in disagreement with our results, as the tea with the highest pH (4.5 for 250 mM Ti-BALDH, Supplementary Materials Figure S1) did not lead to the thickest membranes, and also the yield decreased according the change in thickness of 30.5% (Supplementary Materials Figure S2 and Table S1). This controversy implies that the second influencing factor, namely the presence of Ti-BALDH itself, gains in importance. It appears that the Ti-BALDH is “poisoning” the microorganisms, when reaching a certain threshold concentration, leading to an inhibited BC production.

**Figure 3:**
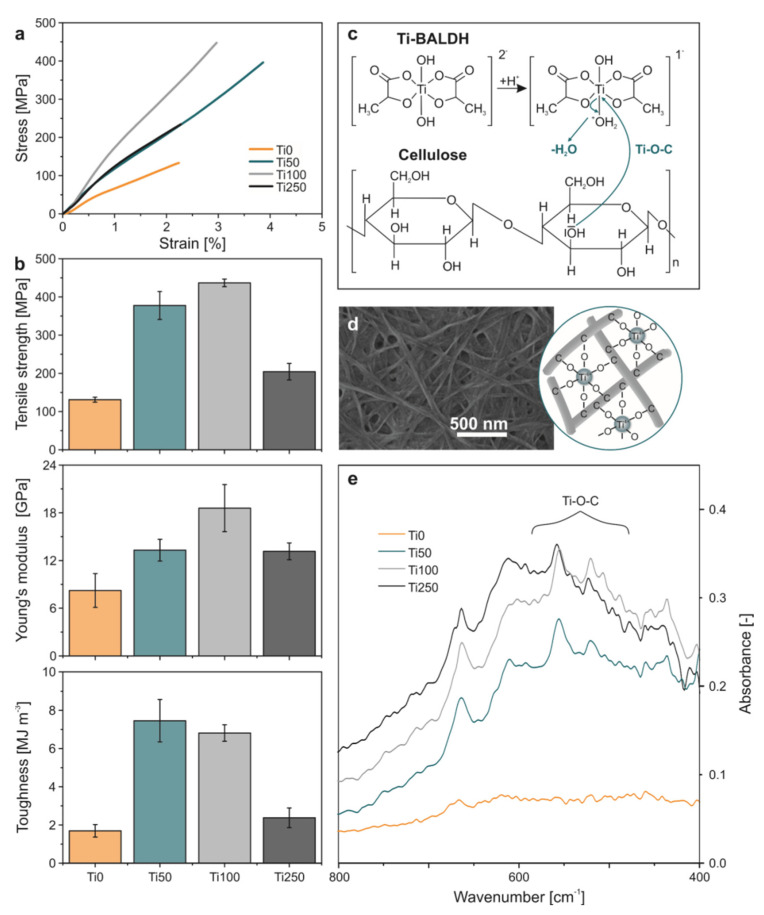
(**a**) Representative stress-strain curves obtained from nanotensile tests of pure BC (Ti0) and BC cross-linked with Ti^4+^ of different content (Ti50, Ti100 and Ti250). (**b**) Corresponding tensile strength, Young’s modulus and toughness values. (**c**) One example of possible Ti-catalysed cross-linking reactions. Analogous to the reaction mechanism, which leads to the cross-linking, all six covalent bonds of the Ti^4+^ can cross-link to cellulose (**d**), as depicted in the cartoon for the SEM image of Ti50. (**e**) The normalized FTIR data reveal a rising band in the region of 400–700 cm^−1^, which can be attributed to Ti-O-C vibrations [20,21].

To this end, in addition to the thickness determination, the membranes were chemically analyzed in terms of their Ti content. Inductively coupled plasma-optical emission spectrometry (ICP-OES) could detect certain amounts of Ti, when the BC pellicles grew in the presence of Ti-BALDH, meaning the Ti became incorporated during the pellicle formation, whereas part of it was removed along with the microorganisms in the post-treatment step (Supplementary Materials Figure S2 and Table S1). In general, a higher Ti-BALDH concentration also resulted in a higher Ti content, whereas the Ti-BALDH uptake appears to follow a rather linear trend from Ti0 to T100, while Ti250 exhibits an over-proportional Ti content (Figure 2a). Investigations on how the Ti content influences the mechanical performance of the membranes were carried out using nanotensile tests. Figure 3a displays the representative stress-strain curves of the four different membranes.

Pure BC (Ti0) membranes reached a tensile strength of 131 ± 7 MPa, a Young’s modulus of 8 ± 2 GPa and a toughness of 1.7 ± 0.3 MJ m^−3^ (Figure 3b and Supplementary Materials Table S2). The measured mechanical performance is in the range of elsewhere reported BC membranes which underwent a similar purification/post-treatment [15,22] and plant-derived cellulose membranes consisting of oriented fibers [6]. In contrast, Ti50 and Ti100 show by far superior mechanical performances, whereas Ti50 outperforms with regard to the toughness (7.5 ± 1.1 MJ m^−3^), while Ti100 outperforms with regard to tensile strength (437 ± 10 MPa) and Young’s modulus (19 ± 3 GPa). The Ti250 membranes again show a reduced mechanical performance, following the trend of sample thickness (Figure 2a). However, their overall mechanical parameters still excel those of Ti0.

In general, the mechanical superiority is assumed to originate from the Ti^4+^-coordinated cross-linking of the BC fibers (Figure 3c), analogous to what was found by Wang et al., who fabricated dopamine-modified alginate beads reinforced by cross-linking via titanium coordination. They reported that the mechanical strength of the beads was enhanced by a factor of ~3.5, upon the addition of Ti-BALDH, as compared to the pure dopamine-modified alginate beads [19]. Our Ti100 membranes also exhibit an enhancement in tensile strength by a factor of ~3.3, as compared to the pure BC membranes. FTIR investigations support the assumption of cross-linking (Figure 3c and Supplementary Materials Figure S6). The membranes which grew in the presence of Ti-BALDH exhibit broad bands in the range of 400 to 700 cm^−1^ and these bands can be assigned to Ti-O-C vibrations [20,21]. Yao et al. also cross-linked BC fibers. They initially broke up the entangled BC fiber network to be able to align the fibers and then subsequently cross-linked them via Cu^2+^ and Fe^3+^ [23]. With these two cross-linkers, they could reach a Young’s modulus of up to 20.2 GPa and 22.9 GPa, respectively. They further imply a dependency of the valence state of the cross-linker, as they also compare their results to BC membranes containing single-valent Na^+^. Along this line, Ti^4+^ should exhibit the highest Young’s modulus. However, the obtained maximum value of 19 ± 3 GPa (Figure 2b) is not superior, leading to the conclusion that the alignment of the fibers likewise plays a major role, similar to what was found for papers composed of V_2_O_5_·*n* H_2_O nanofibers [24,25]. In that case, the alignment of the nanofibers led to an increase in Young’s modulus from 4.8 GPa to ~24 GPa. Analogous to the Youngs’ modulus, Yao et al. report about the valence state-dependency of the tensile strength. They reach values as high as 317 MPa and 357.5 MPa for Cu^2+^ and Fe^3+^, respectively [23], which is also in the range of plant-derived aligned cellulose [6]. These values are inferior to the performance of the Ti^4+^-cross-linked BC Ti100 (437 ± 10 MPa), in spite of the difference in fiber arrangement. Therefore, in terms of mechanical strength, the pronounced impact of cross-linking with multivalent ions outperforms the influence of alignment, making the latter step redundant.

Taking together all the investigated parameters, specifically thickness, Ti uptake, FTIR data and mechanical performance of BC membranes, we conclude that a Ti-BALDH concentration of less than 100 mM is required for proper cellulose membrane formation. Below this value, the cellulose fibers grow smoothly with simultaneous formation of cross-links between them, yielding thick membranes with high mechanical performance due to an effective stress distribution enabled by the uniformly distributed cross-linked fibers within the structure. By contrast, at concentrations above 100 mM (i.e., sample Ti250) much thinner membranes are obtained as a consequence of impeded cellulose fiber growth and/or the occurrence of less fiber branching. Furthermore, the normalized FTIR data on samples Ti100 and Ti250 reveal a similar extent of Ti-C-O bond formation, suggesting the presence of Ti-ions that do not participate in the cross-linking between the fibers. This assumption is further supported by the observation of the lowest mechanical performance for the Ti250 samples, as compared to the other Ti-containing membranes.

## 4. Conclusions

When accurately dosed, Ti-BALDH is not only a powerful tool to boost the pH-driven BC growth rate by more than one order of magnitude, but also enables the eco-friendly, one-pot fabrication of high strength BC membranes, which mechanically outperform their natural counterpart by up to 400%. Thus, these BC membranes of outstanding mechanical performance could find application as separators in flexible electronics, membranes for water purification, tissue engineering for humans or even as artificial skin and ligaments for robotic devices.

## Supplementary Materials

The following are available online at https://www.mdpi.com/1996-1944/13/12/2838/s1, Figure S1: Mixing the fermented Kombucha stock solution with deionized water or Ti-BALDH solution increases its pH. These pH values stay mostly constant over the 7 days of growth, during which the pellicle forms, Figure S2: (a) Photograph of Kombucha mixed with DI water (Ti0) and Ti-BALDH solution of different concentrations (Ti50, Ti100 and Ti250) as well as of the resulting membranes before and after the post-treatment. (b) Thickness and Ti content as a function of Ti-BALDH concentration in the solutions. Even though the reduction of Ti-content also contributes to the decrease of measured thickness, the total amount of Ti inside the membranes is too little to have a significant contribution. The main contribution comes from the removal of the microorganisms, Figure S3: Drying the samples free-standing at room temperature was achieved by placing the rectangular pellicles over the openings of plastic boxes dimension (3 cm × 3 cm). The drying speed strongly depended on the thickness of the samples and took up to one week, Figure S4: Inductive thickness measurements were performed with a W1T3 of Hottinger Baldwin Messtechnik HBM. The device is able to convert a vertical distance into a voltage. After calibration, 1 µm equals 1 V in the setup. The inductive thickness measurement is a non-destructing method. The thickness of the samples can be measured at multiple positions without damaging the samples and still be used for the actuation measurement, Figure S5: In order to investigate the membranes’ microstructure and thickness using SEM, samples were prepared by cutting the part of the fractured samples investigated using a nanotensile test. The figure shows a piece of self-adhesive aluminum tape bent by 90° with the samples attached. The fractured part of the membranes’ cross-section is pointing upwards. All samples were sputtered with 1.5 nm Ir (EM ACE600, Leica) to make them conductive for SEM investigation, Figure S6: The full spectra of normalized FTIR data for all investigated samples. The absorbance signal was normalized by the intensity of the band at 2100 cm^−1^ present in all samples. The origin of the peaks indicated by * is not yet fully clear, most probably they result from overtone and combination vibrations of the cellulose molecules. ** Peak at ~900 cm^−1^ is a signature of amorphous cellulose, Table S1: Ti content and thickness of the as-prepared (contain sugar and microorganisms) and post-treated (free of microorganisms) bacterial cellulose membranes, Table S2: Mechanical properties obtained using nanotensile tests. For each sample at least 15 probes, obtained from different batches, were investigated. The standard deviation was determined from 10 different data sets. Please note that these nanotensile tests were conducted on Ti0 membranes, which were grown for more than 7 days to achieve a thickness allowing for easier sample handling. The thickness was similar to the thickness of Ti50 and Ti100 samples.
