# Supplementary material for: Fast-Growing Bacterial Cellulose with Outstanding Mechanical Properties via Cross-Linking by Multivalent Ions
Source: Materials (Basel). 2020 Jun 24;13(12):2838. doi: 10.3390/ma13122838 (PMC7344470; doi:10.3390/ma13122838)
Supplement: Supplementary file 1 [file materials-13-02838-s001.pdf]

materials

MDPI

Supplemental Materials

Fast-Growing Bacterial Cellulose with Outstanding Mechanical Properties via Cross-Linking by Multivalent Ions

Andrea Knöller, Marc Widenmeyer, Joachim Bill and Zaklina Burghard

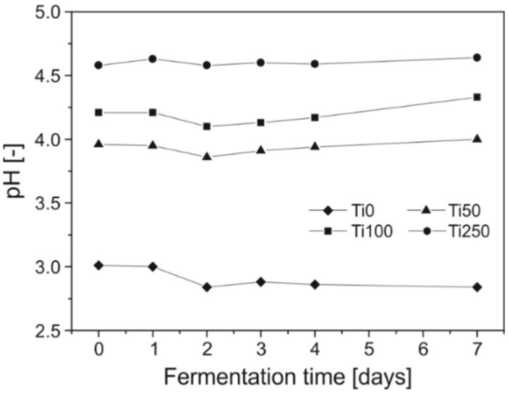

Figure S1. Mixing the fermented Kombucha stock solution with deionized water or Ti-BALDH solution increases its pH. These pH values stay mostly constant over the 7 days of growth, during which the pellicle forms.

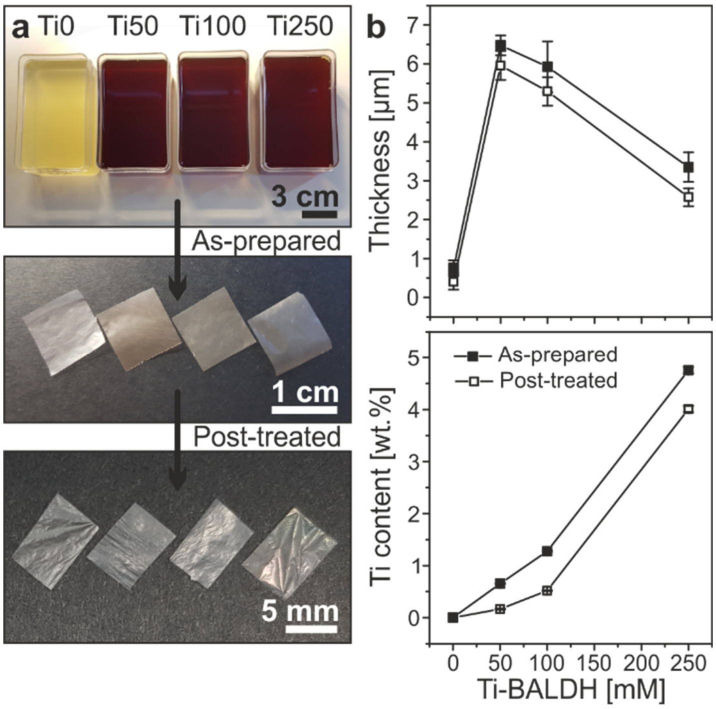

Figure S2. (**a**) Photograph of Kombucha mixed with DI water (Ti0) and Ti-BALDH solution of different concentrations (Ti50, Ti100 and Ti250) as well as of the resulting membranes before and after the post-treatment. (**b**) Thickness and Ti content as a function of Ti-BALDH concentration in the solutions. Even though the reduction of Ti-content also contributes to the decrease of measured thickness, the total amount of Ti inside the membranes is too little to have a significant contribution. The main contribution comes from the removal of the microorganisms.

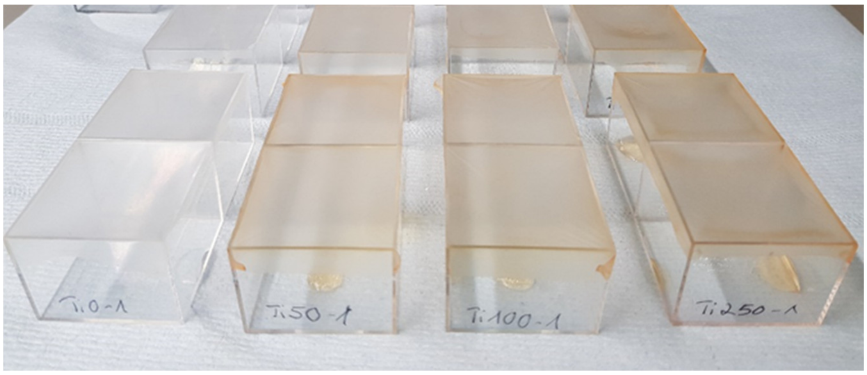

Figure S3. Drying the samples free-standing at room temperature was achieved by placing the rectangular pellicles over the openings of plastic boxes dimension (3 cm × 3 cm). The drying speed strongly depended on the thickness of the samples and took up to one week.

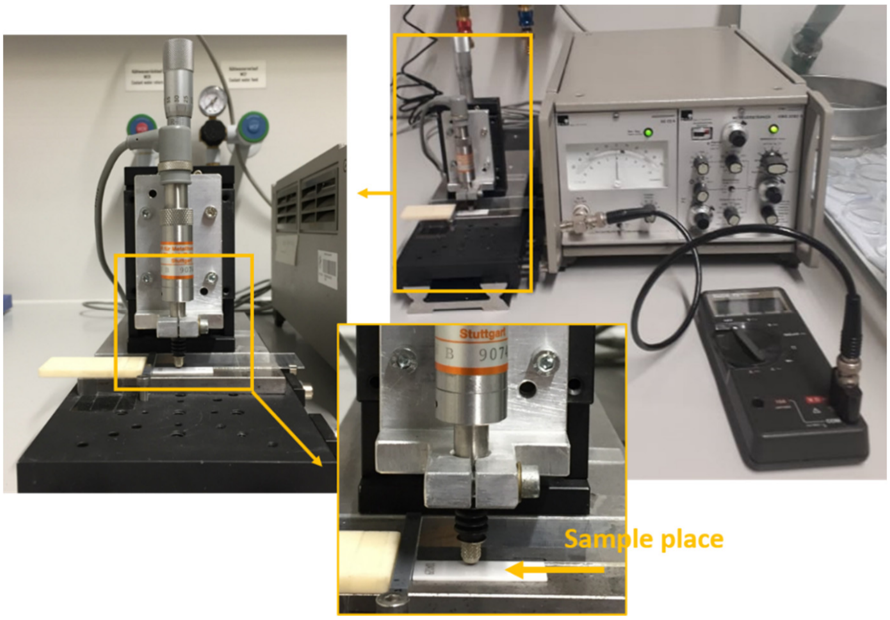

Figure S4. Inductive thickness measurements were performed with a W1T3 of Hottinger Baldwin Messtechnik HBM. The device is able to convert a vertical distance into a voltage. After calibration, 1 μm equals 1 V in the setup. The inductive thickness measurement is a non-destructing method. The thickness of the samples can be measured at multiple positions without damaging the samples and still be used for the actuation measurement.

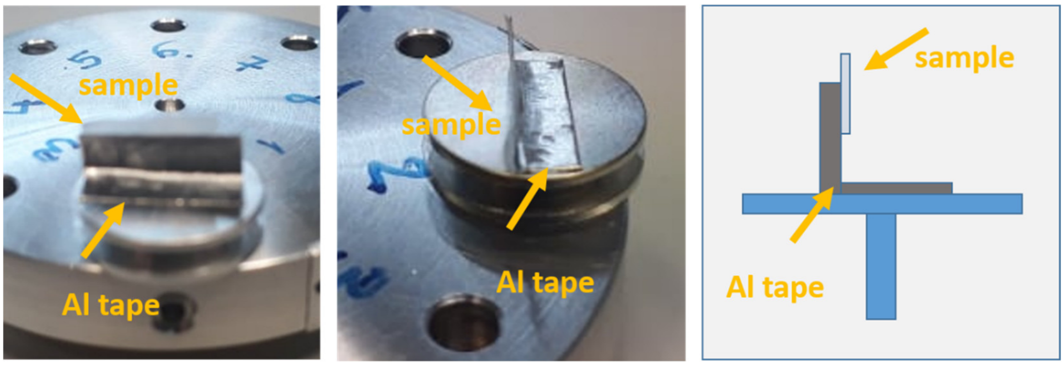

Figure S5. In order to investigate the membranes' microstructure and thickness using SEM, samples were prepared by cutting the part of the fractured samples investigated using a nanotensile test. The figure shows a piece of self-adhesive aluminum tape bent by 90° with the samples attached. The fractured part of the membranes' cross-section is pointing upwards. All samples were sputtered with 1.5 nm Ir (EM ACE600, Leica) to make them conductive for SEM investigation.

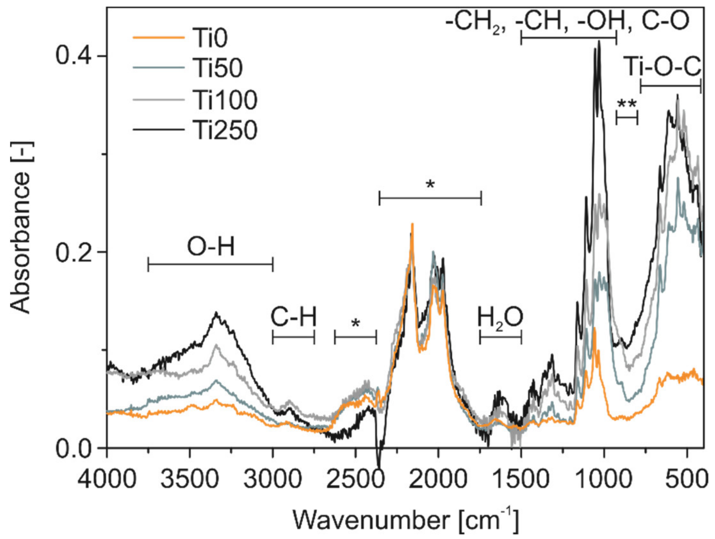

Figure S6. The full spectra of normalized FTIR data for all investigated samples. The absorbance signal was normalized by the intensity of the band at 2100 cm^{-1} present in all samples. The origin of the peaks indicated by * is not yet fully clear, most probably they result from overtone and combination vibrations of the cellulose molecules. ** Peak at ~900 cm^{-1} is a signature of amorphous cellulose.

Table 1. Ti content and thickness of the as-prepared (contain sugar and microorganisms) and post-treated (free of microorganisms) bacterial cellulose membranes.

		Ti0	Ti50	Ti100	Ti250
Ti-content (wt.%)	As-prepared	0	0.66 ± 0.01	1.28 ± 0.02	4.76 ± 0.06
	post-treated	0	0.17 ± 0.01	0.52 ± 0.01	4.01 ± 0.06
Reduction (%)		0	74.8	59.4	15.7
Thickness (µm)	As-prepared	0.9 ± 0.1	6.6 ± 0.2	6.3 ± 0.5	3.7 ± 0.4
	post-treated	0.4 ± 0.2	6.0 ± 0.4	5.3 ± 0.4	2.6 ± 0.2
Reduction (%)		53.5	9.7	15.6	30.5

Table 2. Mechanical properties obtained using nanotensile tests. For each sample at least 15 probes, obtained from different batches, were investigated. The standard deviation was determined from 10 different data sets. Please note that these nanotensile tests were conducted on Ti0 membranes, which were grown for more than 7 days to achieve a thickness allowing for easier sample handling. The thickness was similar to the thickness of Ti50 and Ti100 samples.

**Sample**	**Tensile Strength (MPa)**	**Young's Modulus (GPa)**	**Ultimate Strain (%)**	**Toughness (MJ m^{-3})**
Ti0	131 ± 7	8 ± 2	2.6 ± 0.7	1.7 ± 0.3
Ti50	378 ± 37	13 ± 1	3.7 ± 0.4	7.5 ± 1.1
Ti100	437 ± 10	19 ± 3	3.1 ± 0.3	6.8 ± 0.4
Ti250	204 ± 22	13 ± 1	2.2 ± 0.3	2.4 ± 0.5

© 2020 by the authors. Submitted for possible open access publication under the terms and conditions of the Creative Commons Attribution (CC BY) license (http://creativecommons.org/licenses/by/4.0/).
